# Evaluation of the therapeutic effects of QuickOpt optimization in Chinese patients with chronic heart failure treated by cardiac resynchronization

**DOI:** 10.1038/s41598-018-22525-0

**Published:** 2018-03-09

**Authors:** Ji Yan, Shu Zhang, Dejia Huang, Xiaolin Xue, Jing Xu, Qianmin Tao, Weize Zhang, Zheng Zhang, Wei Hua, Yanchun Liang, Baopeng Tang, Wei Xu, Geng Xu, Xuejun Ren, Jingfeng Wang, Tao Guo, Shaobin Jia, Yugang Dong, Hong Jiang, Guosheng Fu, Liguang Zhu, Lin Chen, Fuli Tian, Feng Ling, Jianmei Li, Xiaoyong Qi, Yinglu Hao, Yutang Wang, Liangrong Zheng, Xiaoqun Pu, Farong Shen, Guangping Li, Hui Li, Fang Peng

**Affiliations:** 1 Department of Cardiology, Anhui Provincial Hospital, No. 17 Lujiang Road, Hefei, 230001 China; 2 Cardiac Arrhythmia center, Fuwai Hospital, Chinese Academy of medical Sciences, No. 167 North Lishi Road, Beijing, 100037 China; 3 Department of Cardiology, West China Hospital, Sichuan University, No. 37 Guoxue lane, Chengdu, 610041 China; 4 Department of Cardiology, The First Affiliated Hospital of Xi’an Jiaotong University, No. 277 Yanta West Road, Xi’an, 710061 China; 5 Department of Cardiology, Tianjin chest hospital, No. 93 Xi’an Road, Tianjin, 300051 China; 6 Department of Cardiology, The First Affiliated Hospital, Zhejiang University, No. 79 Qingchun Road, Hangzhou, 310003 China; 7 Department of Cardiology, The General Hospital of Lanzhou Military, No. 98 Xiaoxihu West Street, Lanzhou, 730050 China; 8 Department of Cardiology, The First Hospital of Lanzhou University, No. 1 Donggang West Road, Lanzhou, 730000 China; 9 Department of Cardiology, The General Hospital of Shenyang Military, No. 83 Wenhua Road, Shenyang, 110840 China; 10 Department of Cardiology, The First Affiliated Hospital of Xinjiang Medical University, No. 137 Liyushan South Road, Urumqi, 830054 China; 11 Department of Cardiology, Nanjing Drum Tower Hospital, The Affiliated Hospital of Nanjing University Medical School, No. 321 Zhongshan Road, Nanjing, 210008 China; 12 Department of Cardiology, The Second Affiliated Hospital of Zhejiang University School of Medicine, No. 88 Jiefang Road, Hangzhou, 310009 China; 13 Department of Cardiology, Beijing Anzhen Hospital, Capital Medical University, No. 2 Anzhen Road, Beijing, 100029 China; 14 Department of Cardiology, Sun Yat-sen Memorial Hospital of Sun Yat-sen University, No. 107 Yanjiang West Road, Guangzhou, 510120 China; 15 Department of Cardiology, The First Affiliated Hospital of Kunming Medical University, No. 295 Xichang Road, Kunming, 650032 China; 16 Department of Cardiology, General Hospital of Ningxia Medical University, No. 804 Shengli South Street, Yinchuan, 750004 China; 17 Department of Cardiology, The First Affiliated Hospital of Sun Yat-sen University, No. 58 Zhongshan Er Road, Guangzhou, 510080 China; 18 Department of Cardiology, Renmin Hospital of Wuhan University, No. 99 Zhangzhidong Road, Wuhan, 430060 China; 19 Department of Cardiology, Sir Run Run Shaw Hospital of Medicine, Zhejiang University, No. 3 Qingchun East Road, Hangzhou, 310020 China; 20 Department of Cardiology, The First Affiliated Hospital of Guangxi Medical University, No. 6 Shuangyong Road, Nanjing, 530021 China; 21 Department of Cardiology, Fujian Provincial Hospital, No. 134 East Street, Fuzhou, 350001 China; 22 The First Department of Cardiology, The 251ST Hospital of PLA, No. 13 Jianguo Road, Zhangjiakou, 075000 China; 23 Department of Cardiology, Hangzhou First People’s Hospital, No. 261 HuanSha Road, Hangzhou, 310006 China; 24 Department of Cardiology, The second people’s Hospital of Yunnan Province, No. 176 Qingnian Road, Kunming, 650021 China; 25 Department of Cardiology, Hebei General Hospital, No. 348 Heping West Road, Shijiazhuang, 050051 China; 26 Department of Cardiology, People’s Hospital of Yuxi City, No. 21 Nieer Road, Yuxi, 653100 China; 27 Department of Cardiology, Chinese PLA General Hospital, No. 28 Fuxing Road, Beijing, 100853 China; 28 Department of Cardiology, Xiangya Hospital Central South University, No. 87 xiangya Road, Changsha, 410008 China; 29 Department of Cardiology, Zhejiang Greentown Hospital, No. 409 Gudun Road, Hangzhou, 310000 China; 30 Department of Cardiology, The Second Hospital of Tianjin Medical University, No. 23 Pingjiang Road, Tianjin, 300211 China; 31 Department of Cardiology, Daqing Oilfield General Hospital, No. 9 Zhongkang Street, Daqing, 163000 China; 32 Department of Cardiology, Shaoxing People’s Hospital, No. 568 Zhongxing North Road, Shaoxing, 312000 China

## Abstract

In this trial, long-term therapeutic effects and clinical improvements in Chinese chronic heart failure patients optimized by QuickOpt or echocardiography were compared for atrioventricular (AV) and interventricular (VV) delay optimizations after cardiac resynchronization therapy (CRT) with pacing (CRT-P) or with pacing and defibrillator (CRT-D) therapy. One hundred and ninety-six subjects (50%) had dilated cardiomyopathy, 108 (27.6%) had ischemic heart disease and 112 (28.6%) were hypertensive and were randomized into QuickOpt (198) or echocardiographic optimization (control) (194) groups at ≤2-weeks post-implantation. Programmed AV/VV delay was optimized at baseline and at 3 and 6 months. Left ventricular end-systolic volume (LVESV), New York Heart Association (NYHA) class, specific activity scale (SAS), and the six-minute walk tests (6MWT) were evaluated by blinded researchers at 12 months. Of the QuickOpt group, LVESV decreased significantly by 24.7% ± 33.9% compared with baseline, while LVESV of Controls decreased by 25.1% ± 36.1% (*P* = 0.924). NYHA class, SAS and 6MWT also improved similarly in both groups at 12 months. Mortality in both groups was not significantly different (11.0% vs 7.6%, *P* = 0.289). However, there was a significant difference in the time required for optimization by QuickOpt compared with echocardiography (3.33 ± 3.11 vs 58.79 ± 27.03 minutes, *P* < 0.000).

## Introduction

Congestive heart failure (CHF) has been estimated to affect more than 26 million people worldwide^1,2^ and is associated with other comorbidities (which include the symptoms of chronic obstructive pulmonary disease, renal impairment, and obstructive sleep apnea), which accounts for about 50% of all readmissions after initial hospitalization for CHF^3,4^. Even today, the prognosis of CHF is poor with studies from the United States (US) and Europe showing that 6-month and 5-year mortality rates remain high at 14% and 45%, respectively^5,6^. Due to its high incidence and mortality, CHF has been deemed to be a worldwide epidemic disease.

Currently, the use of cardiac resynchronization therapy (CRT)^7–11^, including defibrillation capability (CRT-D) or with pacing only (CRT-P), is recommended for patients with heart failure who have left ventricular dysfunction with a left ventricular ejection fraction of ≤35%, on recommended medications as per consensus guidelines for the diagnosis and treatment of CHF.

In China, nearly 3 million CHF patients require CRT-P or CRT-D devices, and proper resynchronization optimization after implantation has become increasingly challenging. Common approaches to define the best AV interval rely on repeated echocardiographic assessments with program adjustments (iterative) and other methods^12,13^, with the aim of interval optimization to reduce left ventricular dyssynchrony and mitral regurgitation. Optimization of both atrioventricular (AV) and ventricular-ventricular (VV) intervals improves the therapeutic effects of CRT^14,15^.

The key to obtaining the optimal AV interval is to synchronize ventricular systolic and diastolic phases. The optimized AV and VV intervals are calculated based on specific medical personnel perceptions and the intracardiac electrogram^16^. The purpose of AV interval optimization is to maximize ventricular preload as well as to allow the mitral valve to close at the proper time. The purpose of VV interval optimization is to make the peak of the left and right ventricular activation meet in the vicinity of the ventricular septum, thereby reducing ventricular dyssynchrony. The effects of this optimization can be assessed by the left ventricular end-systolic volume (LVESV) and left ventricular end-diastolic volume (LVEDV), New York Heart Association class (NYHA), 6-minute walk test (6MWT) and specific activity scale (SAS) according to the US guidelines for CHF diagnosis and therapy^17^.

The QuickOpt algorithm (St. Jude Medical; St. Paul, MN, USA) uses the timing cycle of the cardiac electrogram to perform rapid interval optimization, with a program for use at routine follow-up. It is a rapid (1–4 min) simple, automatic determination of optimal AV/VV delays. Compared with echocardiographic optimization, previous studies have demonstrated that the Aorta Velocity Time Index (AVTI) by QuickOpt optimization and the maximum AVTI by echocardiographic optimization are significantly correlated, with a correlation coefficient of 0.96–0.98^16^.

Small sample sizes and short evaluation periods in existing studies have mainly prevented unequivocal acceptance of the results of CRT with QuickOpt optimization in China. To compare QuickOpt and echocardiographic optimization in the short and long-term, including the clinical outcomes of the two methods across China, we designed a multicenter, prospective, randomized, double blinded, parallel controlled trial.

The main aims of our trial were to compare the therapeutic effects of optimization by QuickOpt or echocardiographic optimization on LVESV and other clinical parameters (NHYA class, SAS, and the 6MWT) in a 31-hospital collaborative cohort of Chinese patients, including adverse events (AE) over one year after CRT-P/D implantation.

## Methods

### Patient selection and study design

All patients were enrolled according to the following criteria for CRT-P/D indications: 1) left ventricular ejection fraction (LVEF) ≤35%; 2) New York Heart Association (NYHA) class III-IV heart failure; 3) QRS complex duration ≥120 ms.

**Figure 1:**
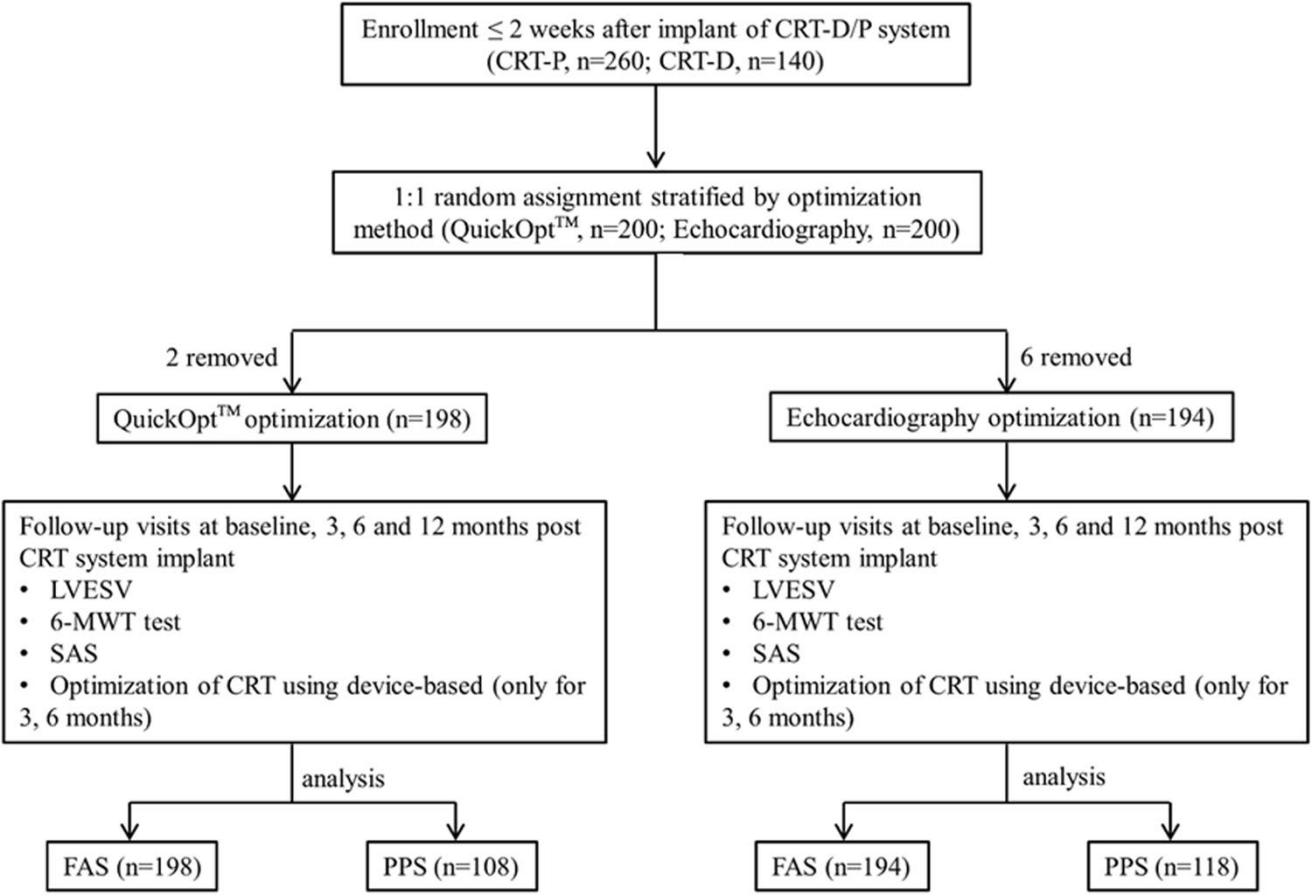
Flow chart for enrolled CHF patients who adopted AV and VV intervals with QuickOpt. CRT-P and CRT-D are device designations; LVESV = left ventricular end-systolic volume; 6-MWT = Six-minute walk test; SAS = specific activity scale; FAS = Full analysis set; PPS = Per protocol set.

Based on the inclusion criteria, 400 patients with CHF in 31 different hospitals from May 2010 to February 2015 were implanted with CRT-P (260 patients) or CRT-D (140 patients) and then they were automatically assigned into the QuickOpt and Echo groups for optimization in a 1:1 ratio by software which linked in all the sites. One hundred and ninety-eight patients were randomized to the QuickOpt group, 194 patients to the echocardiography group after two weeks implantation, and 8 declined randomization because they could not follow up to the end of the research period. We performed CRT-P/D interval optimization during the first two weeks, at 3 months (±2 weeks) and 6 months (±2 weeks) after implantation, and programmed the optimal AV/VV interval in the CRT-P/D (Fig. 1).

Patients who gave informed consent for the study were implanted with CRT-P (Frontier II 5596) or CRT-D (Epic + HF V350, Atlas + HF V341, Atlas II HF V365, Atlas II + HF V366, Atlas II + HF V367, Promote 3107–30, Promote 3107–36) and lead systems (St. Jude Medical, USA) and received optimal medical therapy.

Patients were excluded if they were suffering from severe sinus bradycardia (sinus rate ≤40 bpm) or had persistent chronic atrial tachyarrhythmia, including atrial fibrillation, atrial flutter and atrial tachycardia, or second- or third-degree AV block, or a QRS complex duration less than 120 ms. Other exclusion criteria were:

- patients <18-years;
- life expectancy <1 year;
- received intravenous-positive inotropic drug treatment;
- participating in other device or drug trials;
- being prepared for cardiac transplantation;
- hypertrophic cardiomyopathy;
- severe aortic or mitral stenosis or regurgitation without valve replacement; -pregnant or lactating women;
- coronary artery bypass surgery, percutaneous coronary intervention or cardiomyoplasty in previous 6 weeks;
- acute coronary syndrome;
- stroke;
- pre-excitation.

### Echocardiography group

The apical four-chamber view was used to measure the mitral inflow velocity profile at the mitral valve to optimize AV delay. The AV delay was initially set between 60 ms and 200 ms, and was varied in 20 ms steps. The EA duration was measured at each AV interval, and the optimal AV delay was defined as the maximal EA duration without truncating the A wave^11^.

To optimize VV delay, an apical five-chamber view was used to determine the velocity-time integral (VTI) of the left ventricular outflow tract. The VV delay was set between −40 ms and +40 ms, with programmed adjustments in 20 ms increments; optimal VV delay resulted in a maximal VTI^11^.

### QuickOpt group

AV/VV delay optimization was performed at the intervals stated above with devices being programmed according to data obtained during QuickOpt optimization, namely optimized sensed AV, paced AV and VV intervals.

### Follow-up

Within 2 weeks after CRT-P/D implantation, we used Merlin 3650 PCS (St. Jude Medical, USA) to implement the QuickOpt algorithm, and separately kept 12-lead ECG data of dual chamber pacing for subsequent analysis (Fig. 1). The follow-up data included echocardiography values (only within 12 months), the 6MWT and SAS data (only at 12 months), the NYHA class at each follow-up, 12-lead ECG (pacing), pacemaker follow-up data, and optimization results after QuickOpt optimization (at 3 and 6 months).

This trial complied with the Declaration of Helsinki. Our protocol was approved by the Research Ethics Committee of all 31 hospitals participating (Anhui Provincial Hospita; Fuwai Hospital, Chinese Academy of medical Sciences; The First Affiliated Hospital of Xi’an Jiaotong University; Tianjin Chest Hospital; The First Affiliated Hospital, Zhejiang University; The General Hospital of Lanzhou Military; The First Hospital of Lanzhou University; The General Hospital of Shenyang Military; The First Affiliated Hospital of Xinjiang Medical University; Nanjing Drum Tower Hospital, The Affiliated Hospital of Nanjing University Medical School; The Second Affiliated Hospital of Zhejiang University School of Medicine; Beijing Anzhen Hospital, Capital Medical University; Sun Yat-sen Memorial Hospital of Sun Yat-sen University; The First Affiliated Hospital of Kunming Medical University; General Hospital of Ningxia Medical University; The First Affiliated Hospital of Sun Yat-sen University; Renmin Hospital of Wuhan University; Sir Run Run Shaw Hospital of Medicine, Zhejiang University; The First Affiliated Hospital of Guangxi Medical University; Fujian Provincial Hospital; The 251^st^ Hospital of PLA; Hangzhou First People’s Hospital; The second people’s Hospital of Yunnan Province; Hebei General Hospital; People’s Hospital of Yuxi City; Chinese PLA General Hospital; Xiangya Hospital Central South University; Zhejiang Greentown Hospital; The Second Hospital of Tianjin Medical University; Daqing Oilfield General Hospital; Shaoxing People’s Hospital) in this trial. The trial register number was NCT01172067 (First received: July 28, 2010; Last updated: July 28, 2016; Last verified: July 2016).

### Evaluation of therapeutic effects

#### Primary efficacy

Comparison of changes in LVESV between the 2 groups, 12 months after implantation. All echocardiography images were collected and measured in one core laboratory to reduce any measurement bias.

#### Secondary efficacy

A blinded researcher evaluated changes in clinical parameters of patients at 12 months, including NHYA class, SAS and the 6MWT. SAS is a simple method to assess body functions.

#### Other efficacy

Safety indicators; AE (any adverse medical events) since the patients signed consent until the end of the study, regardless of the cause. Severe AEs that occurred during the course of the clinical study, such as the need for hospitalization, prolonged hospitalization, disability affecting patient’s work, life threatening events or death.

AE related to CRT-P/D included, but were not limited to:

- worsening heart failure;
- pacing system infection;
- elevated pacing threshold;
- lead dislocation;
- lead fracture;
- lead insulation damage;
- death.

Each center had a physician specifically responsible for collecting data and then filling in the Electronic Data Capture (EDC). The data were reliable and every record of death and hospitalization was documented.

The report of death incidence consisted of two procedures. Once informed of a death, physicians evaluated the cause and reported it to the ethics department in the hospital within 24 hours. Then the ethics department reported to the China Food and Drug Administration (CFDA). In the meanwhile, physicians filled the death table in the electronic CRF within 3 days, and kept the original medical record as the initial document.

### Statistical analysis

We hypothesized that 12 months after implantation, the improvement in LVESV in the QuickOpt group would equal that of the echocardiography group. As the presumed lower limit value was −10%, the assumed significant difference was 0.05 (1-sided), the detection efficacy was 80%, the overall standard deviation was 0.35, and the difference between the two group averages was 0.1, we needed 306 cases in total and 153 cases for each group. Taking into account 20% loss of cases due to death, heart transplantation, loss to follow-up and other causes, we finally enrolled 200 cases in each group, with a total cohort size of 400 cases. For primary efficacy, the difference between the two types of optimization was evaluated by Student’s *t*-test.

For secondary efficacy, the improvements in the NYHA class and SAS evaluation between the two types of optimization were compared with a full analysis set (FAS) and a Cochran-Mantel-Haenszel test. If the results of a chi-square test yielded a homogeneous distribution, the Fisher exact test was used. The improvement in the 6MWT between the two optimization methods was also compared with FAS and any significant differences evaluated by means of a *t*-test. If the result was an obvious non-normal distribution and heterogeneity of variance according to the distribution of final data, we adopted one of the following non-parametric statistics: Wilcoxon rank sum test or the Kolmogorov-Smirbov test. The FAS was an ideal group of participants as close to the Intention To Treat (ITT) principle as possible, which included nearly all subjects after randomization. The data were analyzed according to the intention-to-treat principle. The per protocol set (PPS) was a subset of the full analysis set, and each subject in this data set showed good compliance without violating the program. In our study, there were 392 cases consistent with the FAS set and 226 consistent with the PPS set.

### Data availability

The datasets used and analyzed during the current study are available from the corresponding author on reasonable request.

## Results

### Clinical data

The research cohort in this trial was comprised of 392 patients with CHF, in which 289 patients were male and 103 female. The mean age of the QuickOpt group was 61.38 ± 11.77 years and the echocardiography group 59.21 ± 11.36 years. 196 patients were diagnosed with dilated cardiomyopathy and 112 patients with hypertension, 47 patients with diabetes and <10 cases of hypertriglyceridemia, valvular heart disease, stroke or paroxysmal atrial fibrillation; however, there were 15 cases of stroke in both groups. In comparison of demographics, baseline data in the two groups showed no significant differences (Table 1). Among them, 27.6% (108) had ischemic heart disease and 72.4% (283) non-ischemic heart disease (Table 2). However, there were more patients with first-degree AV block in the QuickOpt than in the echocardiography group, and the mean LVEF was higher in the QuickOpt than in the echocardiography control patients (Table 2). There were no significant differences in the duration of heart failure, heart rate, QRS duration, different types of bundle branch block, or mean LVEDDs between the two groups (Table 2).

**Table 1:** Demographics and baseline characteristics of enrolled patients.

Index	QuickOpt group (n = 198)	Echocardiography group (n = 194)	*P*-value
Gender	198 (0)	194 (0)	0.494
Male	149 (75.25%)	140 (72.16%)	
Female	49 (24.75%)	54 (27.84%)	
Age (year)	61.38 ± 11.77	59.21 ± 11.36	0.065
Height (cm)	167.10 ± 7.89	166.33 ± 6.43	0.293
Weight (kg)	64.82 ± 12.53	63.16 ± 10.19	0.151
BMI (kg/m^2^)	23.08 ± 3.37	22.77 ± 3.05	0.338
Systolic blood pressure (mmHg)	115.24 ± 17.18	116.98 ± 16.03	0.301
Diastolic blood pressure (mmHg)	72.70 ± 10.71	73.53 ± 10.00	0.428
*Previous medical history (PMH)*			
Hypertension	60 (30.30%)	52 (26.80%)	0.503
Hypertriglyceridemia	6 (3.03%)	2 (1.03%)	0.284
Diabetes	25 (12.63%)	22 (11.34%)	0.757
Valvular heart disease	5 (2.53%)	0 (0.00%)	0.061
Dilated cardiomyopathy	99 (50.00%)	97 (50.00%)	1.000
Stroke	9 (4.55%)	6 (3.09%)	0.600
Paroxysmal atrial fibrillation	2 (1.01%)	7 (3.61%)	0.102

Note: BMI = Body Mass Index

**Table 2:** Comparison of the two groups of patients with cardiovascular disease.

Index	QuickOpt group (n = 198)	Echocardiography group (n = 194)	*P*-value
Etiology of heart failure N = 391(1)	198 (0)	193 (1)	1.000
Ischemic	55 (27.78%)	53 (27.46%)	
Non-ischemic	143 (72.22%)	140 (72.54%)	
Heart failure N	177 (21)	167 (27)	0.825
Duration of heart failure (days, median (Q1, Q3))	809.00 (157.50; 1577.50)	641.00 (140.00; 1709.00)	0.485
Heart rate (bpm)	77.15 ± 14.22	75.24 ± 14.38	0.187
QRS duration (ms)	154.03 ± 25.06	151.75 ± 23.14	0.351
First -degree AV block N (%)	34 (17.17%)	17 (8.76%)	0.037
Bundle branch block (BBB) N			0.228
Left BBB	125 (63.13%)	120 (61.86%)	
Right BBB	12 (6.06%)	4 (2.06%)	
Intraventricular conduction delay	11 (5.56%)	11 (5.67%)	
Others N	50 (25.25%)	59 (30.41%)	
LVEF (%)	28.74 ± 5.13	27.30 ± 6.04	0.011
LVEDD (mm)	72.76 ± 9.18	73.83 ± 9.89	0.270

Note: N = number; AV = atrioventricular block; LVEF = left ventricular ejection fraction; LVEDD = left ventricular end-diastolic volume. Q1 and Q3 = interquartile range.

### Comparison of CRT implantation methods and optimization

In the QuickOpt optimization group, 99 patients received CRT-Ps and 99 patients CRT-Ds. In the echocardiography control group, 95 patients received CRT-Ps and 99 patients CRT-Ds. 99.5% of the CRTs were implanted on the left side and the leads were placed through the left subclavian vein. The positions of the electrodes in the posterior lateral veins in the QuickOpt and echocardiography control group were 50.0% and 47.9%, respectively and in the lateral veins 38.1% and 38.4%, respectively. The depth of the electrodes in the veins middle segment accounted for 65.2% and 72.2%, respectively, and the depth of electrodes in the veins distal segment 31.3% and 23.7%, with the depth in the proximal segment being 3.5% and 4.1%. Therefore, no significant differences were observed between the QuickOpt optimization group and the echocardiography control group, with regard to the device implantation and leads positions (Supplementary Table 1). Supplementary Table 2 lists the baseline conditions of the patients. There were no significant differences between the two groups in the positions of the right atrial, right ventricular and left ventricular leads, as well as in the 3 month, 6 month and 12 month program controls (data not shown).

A comparison of the optimization process, optimization results and programmed control data in the two groups (shown in Table 3), revealed that the optimization time of the QuickOpt group at baseline, 3 months and 6 months required a median time of 2 minutes, whereas the echocardiography group had a mean time of 54.25–58.79 minutes, and the median time was 43.50–55.00 minutes.

**Table 3:** Optimization at baseline, 3 months and 6 months after operation (FAS).

Index	Baseline		3 months		6 months	
	QuickOpt group	Echocardiography group	QuickOpt group	Echocardiography group	QuickOpt group	Echocardiography group
**Optimization process N = 386**	197 (1)	189 (5)	169 (29)	164 (30)	154 (44)	154 (40)
**Time required (min)**						
Mean ± SD	3.33 ± 3.11	58.79 ± 27.03*	3.11 ± 2.81	54.25 ± 27.89*	3.06 ± 2.69	54.70 ± 27.93*
Median (Q1,Q3)	2.00 (1.00;5.00)	55.00* (40.00;82.0)	2.00 (1.00;5.00)	43.50* (30.00;80.00)	2.00 (1.00;4.00)	50.00* (30.00;80.00)
**Optimization results**						
PV interval (ms)	169.85 ± 15.13	162.20 ± 21.16*	168.53 ± 16.84	159.18 ± 23.49*	169.61 ± 14.68	159.81 ± 24.84*
AV interval (ms)	122.98 ± 16.21	126.46 ± 20.15	121.29 ± 16.16	123.58 ± 19.50	120.59 ± 13.60	122.32 ± 20.66
VV interval (ms)	32.53 ± 16.99	27.32 ± 15.72*	34.32 ± 16.35	30.92 ± 18.45	36.47 ± 16.74	32.48 ± 16.08
Sequence of results N	198(0)	189 (5)	169 (29)	165 (29)	155 (43)	154 (40)
Left ventricle first	138 (69.70%)	102 (53.97%)*	118 (69.82%)	100 (60.61%)*	117 (75.48%)	99 (64.29%)*
Right ventricle first	20 (10.10%)	9 (4.76%)*	11 (6.51%)	6 (3.64%)*	11 (7.10%)	6 (3.90%)*
Simultaneously	40 (20.20%)	78 (41.27%)*	40 (23.67%)	59 (35.76%)*	27 (17.42%)	49 (31.82%)*
**Programmed controls**						
PV interval (ms)	169.39 ± 15.63	162.13 ± 21.12*	167.82 ± 16.87	159.30 ± 23.50*	169.23 ± 15.18	159.61 ± 24.80*
AV interval (ms)	122.73 ± 16.91	126.32 ± 20.19	120.94 ± 16.44	123.70 ± 19.42	120.32 ± 13.79	123.03 ± 23.00
VV interval (ms)	31.97 ± 16.99	27.75 ± 15.46*	34.73 ± 16.22	31.53 ± 17.99	36.69 ± 16.65	32.82 ± 15.87
Sequence of results N	198(0)	190 (4)	169 (29)	165 (29)	155 (43)	154 (40)
Left ventricle first	138 (69.70%)	102 (53.68%)*	121 (71.60%)	102 (61.82%)*	116 (74.84%)	99 (64.29%)*
Right ventricle first	20 (10.10%)	9 (4.74%)*	9 (5.33%)	5 (3.03%)*	11 (7.10%)	6 (3.90%)*
Simultaneously	40 (20.20%)	79 (41.58%)*	39 (23.08%)	58 (35.15%)*	28 (18.06%)	50 (32.47%)*

^*^Significant difference compared to QuickOpt group *P* < 0.05 N = number; SD = standard deviation; PV = paced atrial- paced ventricular; AV = sensed atrial to paced ventricular; VV = ventricular-ventricular; Q1 and Q3 = interquartile range.

Optimization results showed that the PV (paced atrial to paced ventricular) interval in the QuickOpt group was significant longer than in the echocardiography group at baseline, 3 months and 6 months (169.85 ± 15.13; 162.20 ± 21.16; 168.53 ± 16.84 vs 159.18 ± 23.49; 169.61 ± 14.68 vs 159.81 ± 24.84 ms). The VV interval in the QuickOpt group was only significantly longer at baseline (32.53 ± 16.99 vs. 27.32 ± 15.72 ms), but there were no significant differences between the QuickOpt and the echocardiography groups in the AV (atrial sense to ventricular pace) interval at any stage.

### Comparison of the therapeutic effects on the two groups

Twelve months after optimization, the LVESV of the QuickOpt group was significantly decreased by 24.7 ± 33.9% compared with baseline, while the LVESV of the control group was decreased by 25.1 ± 36.1%, *P = *0.924. The LVEDV of the 2 groups over 12 months showed significant improvement but there was no significant difference between the two optimization groups. The LVEF was significantly increased 12 months after optimization in the QuickOpt and control groups, being 38.4 ± 44.3% and 38.4 ± 44.8%, respectively, which demonstrates that the primary efficacy of the QuickOpt optimization was not inferior to the efficacy of the echocardiography (Table 4).

**Table 4:** Comparison of left ventricular end-diastolic volume (LVESV), left ventricular end-diastolic volume (LVEDV) and left ventricular ejection fraction (LVEF) improvements in the two groups 12 months after implantation (PPS).

	QuickOpt group (n = 108)	Echocardiography group (n = 118)	*P*-value
LVESV (Mean ± SD)			
Baseline	165.60 ± 66.89	172.25 ± 66.85	0.456
12 months after implantation	125.42 ± 82.28*	127.96 ± 79.59*	0.814
LVESV improvements (mL)	39.80 ± 62.30	44.75 ± 68.99	0.602
LVESV reduction (%)	24.69 ± 33.86	25.13 ± 36.06	0.924
LVEDV(Mean ± SD)			
Baseline	232.75 ± 84.62	238.88 ± 79.81	0.576
12 months after implantation	200.81 ± 102.99*	196.40 ± 94.00*	0.737
LVEDV improvements (mL)	33.53 ± 78.51	42.48 ± 79.15	0.396
LVEDV reduction (%)	13.56 ± 31.55	16.44 ± 31.04	0.492
LVEF (Mean ± SD)			
Baseline	30.70 ± 7.79	29.12 ± 6.80	0.106
12 months after implantation	40.54 ± 10.89*	39.29 ± 11.84*	0.413
LVEF improvements (%)	10.15 ± 10.99	10.16 ± 11.11	0.991
LVEF increase by (%)	38.44 ± 44.29	38.44 ± 44.83	1.00

^*^Compared to baseline, **LVEF increase by (%) over baseline *P* < 0.05; PPS = Per Protocol Set.

In addition, we found that the NYHA class for QuickOpt optimization patients was significantly improved 3, 6 and 12 months after CRT-P/D implantation. The number of NYHA class III and IV patients was significantly reduced, but there was no significant difference in improvement between the QuickOpt and echocardiography control groups (Table 5).

**Table 5:** Comparison of differences between QuickOpt group and echocardiography group before and after optimization in NYHA classification (FAS).

	N (missing)	NYHA					*P*-value
		I	II	III	IV		
Baseline	QuickOpt group	198 (0)	1 (0. 51%)	0 (0.00%)	160 (80.81%)	37 (18.69%)	0.423
	Echocardiography group	193 (1)	0 (0.00%)	1 (0.52%)	162 (83.94%)	30 (15.54%)	
3 months	QuickOpt group	182 (16)	21 (11.54%)*	101 (55.50%)*	59 (32.42%)*	1 (0.55%)*	0.552
	Echocardiography group	176 (18)	19 (10.80%)^△^	110 (62.50%)^△^	46 (26.14%)^△^	1 (0.57%)^△^	
6 months	QuickOpt group	168 (30)	22 (13.10%)*	108 (64.29%)*	36 (21.43%)*	2 (1.19%)*	0.586
	Echocardiography group	169 (25)	26 (15.38%)^△^	114 (67.46%)^△^	27 (15.98%)^△^	2 (1.18%)^△^	
12 months	QuickOpt group	153 (45)	35 (22.88%)*	90 (58.82%)*	25 (16.34%)*	3 (1.96%)*	0.142
	Echocardiography group	163 (31)	36 (22.09%)^△^	111 (68.10%)^△^	15 (9.20%)^△^	1 (0.61%)^△^	

^*^Significant difference compared to baseline in the QuickOpt group *P* < 0.05, ^Δ^significant differences in the echocardiographic group, *P* < 0.05.FAS = Full analysis set; N = number, Missing = Not Done.

The SAS evaluation scores in both the QuickOpt and echocardiography groups were not significantly different throughout the study.

Finally, twelve months after CRT-P/D implantation, the 6MWT improved in both groups. However, there was no significant difference, which was consistent with the secondary efficacy of QuickOpt optimization being not inferior to echocardiography optimization (Table 6).

**Table 6:** Comparison of differences in modified SAS and 6MWT evaluation between the QuickOpt and the echocardiography groups before and 12 months after optimization (FAS).

		After 12 months		*P*-value*
		QuickOpt group	Echocardiography group	
Modified SAS	N (baseline/12 months)	198/153	193/163	
	Improved	107 (69.93%)	130 (79.75%)	0.085
	No change	40 (26.14%)	31 (19.02%)	
	Deteriorated	6 (3.92%)	2 (1.23%)	
6MWT	N (baseline/12 months)	197/133	193/154	
	Improvement (meters)	112.70 ± 139.33	129.33 ± 112.49	0.274

*(QuickOpt vs echocardiography at 12 months)

FAS = Full analysis set; SAS = specific activity scale; 6MWT = Six minute walk test; N = number.

### Comparison of the incidence of adverse events and mortality rates between the two groups

There was no significant difference in the incidence of device-related AE between the QuickOpt and echocardiography groups (Table 7), although there were 27 SAEs in the QuickOpt group and 19 in the echocardiography group. Three AEs were associated with the device in the QuickOpt group, 1 with increased LV pacing threshold, 1 electrode dislocation, and 1 junctional rhythm and low blood pressure. In the echocardiography group, there was 1 patient with an increased LV pacing threshold, and 1 patient with exacerbation of heart failure. There was no significant difference in mortality rates between the QuickOpt and echocardiography group (11.0% vs 7.6%, *P* = 0.289) (Table 7).

**Table 7:** Summary of adverse events (AE).

Item	QuickOpt group			Echocardiography group			*P*-value
	Cases	Numbers	Percentage	Cases	Numbers	Percentage	
Total adverse events	33	31	15.7% (31/198)	25	24	12.4% (24/194)	0.385
Adverse events related to device^*^	3	3	1.5% (3/198)	2	2	1.0% (2/194)	1.000
Severe adverse events	27	25	12.6% (25/198)	19	19	9.8% (19/194)	0.425
Mortality rates		21	11.0% (21/191)		14	7.6% (14/185)	0.289

^*^AE events related to the device: “affirmative relevant”, “likely to be relevant”, “may be related”.

## Discussion

In the present study, we confirmed in a large-scale clinical trial using QuickOpt optimization against the standard echocardiographic method in Chinese congestive heart failure patients treated by cardiac resynchronization, that the hemodynamic efficacy of QuickOpt optimization was not inferior to the echocardiographic method. These results were achieved by using QuickOpt in a time as rapidly as 3 min compared with 56 min by echocardiography (*P* < 0.05).

The AV/VV interval optimizations, assessed blindly, were associated with improvement in heart function and a reduction in mitral regurgitation. Ritte *et al*.^18^ reported that the optimal AV delay was 100–120 ms. We found using QuickOpt, that the optimized AV delay was 122.98 ± 16.21 ms at baseline, 121.29 ± 16.16 ms at 3 months and 120.59 ± 13.60 ms at 6 months, which were not significantly different from echocardiographically determined intervals. By extending Left Ventricular Filling Time (LVFT) and thus raising LVEF, mitral valve regurgitation was reduced. The two methods together with programmed control of AV/PV and VV have improved heart function (NYHA scores) and left ventricular end-systolic volume (LVESV) (Table 4). The 6MWT and SAS scores were also significantly improved at 12-months compared with baseline.

The PV delay time exhibited a significant difference between QuickOpt and echocardiography optimizations at baseline, 3 months and 6 months, due to the optimal AV delay defined as the maximal EA duration without truncating the A wave, combined with the optimal VV delay producing a maximal LV outflow velocity-time integral. This difference in PV was also seen in the small cohort study of Wang *et al*.^10^ without adverse effects on the parameters of LV function.

The significant difference in the optimization time by QuickOpt compared with echocardiography (3 min vs 56 min, *P* < 0.05) is a valuable clinical benefit with this methodology, which is non-inferior to echocardiography and needing circa 50 min less time than echocardiographic analysis. In a busy medical environment, this time saving allows physicians and technologists to reallocate echocardiography services for other patients.

Recent clinical studies using QuickOpt have unequivocally demonstrated that the Aorta Velocity Time Index (AVTI) determined by QuickOpt and the maximum AVTI by echocardiography optimization are significantly correlated (correlation coefficient r = 0.96–0.98)^10^. During echocardiographic optimization, optimal AV/VV delays are determined by the mitral inflow velocity and left ventricular outflow tract velocity profiles, which are measured from Doppler signals. The ideal AV delays show separation of the E and A waves on transmitral inflow Doppler signals^19^ while QuickOpt optimization is an algorithm which rapidly determines optimal AV and VV intervals based on heart electrical activity as measured by intracardiac electrography^20^. This approach should ensure that both intrinsic wave front and pacing stimuli arrive at the ventricular septum simultaneously to optimize the AV/VV delays. Thus, the use of QuickOpt optimization is easier to implement and far less time consuming^10^. Wang’s group also suggested that those patients who do not respond to CRT should receive echocardiographic optimization, which may result in a better hemodynamic outcome.

The other major aspect of our study is that we were able to demonstrate that the efficacy of QuickOpt optimization on parameters of cardiac function was comparable with that of the echocardiographic method. Further, LVEF was significantly increased at 12 months in both optimization groups, being 38.4 ± 44.3% and 38.4 ± 44.8%, respectively. Wang and colleagues^10^ reported similar findings in their smaller number of patients when assessed at 12 months after optimization. In our study, we clearly demonstrated that there were no significant differences in LVESV, LVEDV and LVEF between the two groups at 12 months (Table 4). Another important point from this trial is that patients from 31 hospitals were followed up at 3, 6 and 12 months, presenting a detailed and robust longitudinal clinical study with clearly effective clinical utility. The follow-up data included echocardiography (within 12 months), the 6MWT assessment (at 12 months), the NYHA class at each follow-up, 12 lead ECG (pacing), pacemaker follow-up data, and optimization results after QuickOpt optimization (at 3 and 6 months).

Finally, it is important to note that there were no significant differences in the incidence of device-related AEs and mortality rates between the QuickOpt and echocardiography groups (Table 7).

## Conclusions

This multi-center and large cohort trial has shown that QuickOpt is a rapid and simple method to carry out optimization of AV and VV delays, which is non-inferior compared to the echocardiographic method in terms of resulting cardiac function and adverse events. For busy cardiology departments this will significantly free up precious clinical time.

## Electronic supplementary material

CONSORT 2010 checklist

protocol

Supplementary Datasets
