# Supplementary material for: Evaluation of the therapeutic effects of QuickOpt optimization in Chinese patients with chronic heart failure treated by cardiac resynchronization
Source: Sci Rep. 2018 Mar 9;8:4259. doi: 10.1038/s41598-018-22525-0 (PMC5844885; doi:10.1038/s41598-018-22525-0)
Supplement: Supplementary file 2 — protocol [file 41598_2018_22525_MOESM2_ESM.pdf]

Project No.:	CR-09-063-AP-HF
Project Name:	QuickOpt™ Chronic Study
Version:	3.0
Date:	20100310

QUICKOPT™ CHRONIC STUDY - Clinical Impact of Cardiac Resynchronization Therapy on Heart Failure Patients With QuickOpt and Echo Optimization

Clinical Investigation Plan (CIP)

SPONSOR REPRESENTED BY

SJM International, Inc.
The Corporate Village Da Vincilaan 11- box F1
B-1935 Zaventem, Belgium
Tel: +32 2 774 69 37 Fax: +32 2 774 69 46

LOCAL SPONSOR

St. Jude Medical (Shanghai) Co. Ltd.
20th floor, Xuhuiyuan Mansion, No.1089,
2nd South Zhongshan Road, Xuhui District, Shanghai, 200030, P.R.C
Tel: (86) 21 23067588 Fax: (86)21 64224838

VICE PRESIDENT CLINICAL OPERATIONS ______________________

DATE ______________________

PRINCIPAL INVESTIGATOR

Name: 1. Zhang Shu, Professor	2. Huang Dejia, Professor
Hospital: Fuwai Hospital, Chinese Academy of Medical Sciences (CAMS)	2. West China Hospital
Department: 1. Diagnosis and Treatment Center of Arrhythmia	2. Department of Cardiovascular Medicine
Address: 1. No.167 North Lishi Road, Xicheng District, Beijing, China	2. No.37, Guoxue Alley, Chengdu, Sichuan, China,
City: 1. Beijing	2. Chengdu, Sichuan
Country: China	
Tel.: 1. (86) 10 88398843	2. (86) 28 85422343
Fax: 1. (86) 10 68334688	2. (86) 28 85582944

SINATURE ______________________

DATE ______________________

2010 - SJM International, Inc.: Any information contained in this plan shall not be reproduced without the written permission of SJM. All rights reserved.

Table of Contents

1 Introduction 5
1.1 Background 5
1.2 Interval Optimization 5
1.3 Left ventricular (LV) remodeling 6
1.4 Assessment of body function 6
2 Study Objectives 7
2.1 Purpose 7
2.1.1 Primary objectives 7
2.1.2 Secondary objective 7
2.2 Study endpoints 7
2.2.1 Primary endpoint 7
2.2.2 Secondary endpoints 7
2.2.3 Additional data: 7
2.3 Screening criteria of patients 8
2.3.1 Inclusion criteria 8
2.3.2 Exclusion criteria 8
2.4 Study design 8
2.4.1 Study Procedures 9
2.5 Devices used 10
2.5.1 CRT-P: 10
2.5.2 CRT-D: 11
2.6 Sample size and duration of the study 11
3 Protocol Description 12
3.1 Enrollment 12
3.2 Baseline visit (within two weeks after implantation) 12
3.2.1 Randomization 12
3.2.2 Baseline follow-up (within two weeks after implantation) 12
3.3 3 & 6-month follow up after implantation 13
3.4 12-month follow up after implantation 14
3.5 Unscheduled follow up 14
3.6 Deviation from protocol 14
3.7 Adverse events 15
3.8 End of study 16
3.9 Death of patients 16
4 Statistics 17
4.1 Sample size 17
4.1.1 Assumptions 17
4.1.2 Calculation of sample size 17
4.2 Statistical method 17
5 Risk and Benefit 18
5.1 Risk 18
5.2 Benefit 18
6 Study Organization 19
6.1 Study management 19

6.1.1 Sponsor 19
6.1.2 Clinical Research Organization (CRO) 19
6.2 Investigators 19
6.2.1 Principal investigator 19
6.2.2 Responsibilities of the investigators 20
6.3 Steering Committee (SC) 20
6.3.1 SC 20
6.4 Ethical basis 21
6.5 Inspection 21
6.5.1 Site initiation: 21
6.5.2 On-site investigation 21
6.5.3 Inspection of supervision authority (CA) 22
6.6 Contacts of study management 22

Appendix A: Abbreviations 23
Appendix B: Bibliography 24

1 Introduction

1.1 Background

The effect of Cardiac Resynchronization Therapy (CRT) on Chronic Heart Failure (CHF) has been recognized internationally. The multi-center clinical study proved that CRT could not only improve the cardiac function of CHF patients with CHF with cardiac dyssynchrony, increase 6-Minute Hall Walk Distance (6MHWD), improve quality of life, reduce admission rate and reverse LV remodeling [1-4], but also significantly reduce mortality of HF patients and improve their clinical prognosis [5-6]. Therefore, the heart failure treatment guidelines developed by European Society of Cardiology (ESC) and American College of Cardiology/American Heart Association (ACC/AHA) in 2005 changed the indications for CRT device implantation from Class IIa to Class I in some CHF patients with cardiac dyssynchrony in succession.

1.2 Interval Optimization

The purpose of this CRT is to reduce even eliminate the AV, VV and intraventricular systolic dyssynchrony through providing appropriate AV interval and VV interval so as to improve the hemodynamic status of CHF patients. The optimization of AV interval improves the effective LV filling in CRT patients [7-8] while the purpose of the optimization of VV interval is to weaken LV dyssynchrony and reduce mitral regurgitation [9-10] and improve some non-responders with only AV interval optimized to responders [11-12]. Therefore, the importance of optimization of AV and VV intervals on the improvement of clinical efficacy in CRT patients has been recognized.

Currently, the commonly used optimization method is echocardiography; however, since the echocardiographic optimization is expensive, time-consuming and with low reproducibility, many patients receive interval optimization only before they are discharged or when they have poor response to CRT so that the clinical efficacy of CRT is adversely affected. In addition, the optimal value of AV interval in patients receiving CRT changes with the time [13-16]. As a result, the AV interval shall be optimized periodically based on the change of conditions in clinical practice to improve the clinical efficacy of CRT.

According to the epidemiology and current indications for CRT device implantation, there are approximately 300,000 CHF patients requiring CRT-P/D implantation in China. In 2009, the total number of implanted CRT-P/Ds was more than 1,000 in China with significant increase trend in annual quantity. Therefore, the post-implantation parameter optimization has become a great challenge in clinical practice.

QuickOpt™ developed by St Medical (SJM) in 2007 provides a rapid interval optimization using intracardiac ECG. That is to say during routine follow-up using programmer, for the optimized AV interval and VV interval calculated according to specific sensing and pacemaker intracardiac ECG, the purpose of AV interval optimization is to try to maximize the ventricular preload and close the mitral valve at appropriate time while the purpose of VV interval optimization is to try to make the wave fronts activated at LV and RV leads meet near interventricular septum, so as to reduce cardiac dyssynchrony.

In comparison with echocardiographic optimization, interval optimization with QuickOpt™ is time saving and economical with good patient compliance. The currently published clinical study results demonstrated that the aortic velocity time integral (AVTI) obtained through QuickOpt™ optimization was significantly associated with the maximum AVTI through echocardiographic

optimization, with the correlation coefficient up to 0.96-0.98 [17]. In addition, both QuickOpt™ optimization and echocardiographic optimization improve the hemodynamics in heart failure patients [18]. The study showed that compared with the real-time three-dimensional echocardiography (RT3DE) method, the LV synchronization and clinical manifestations of patients before and after interval optimization were significantly improved with the QuickOpt™ interval optimization [19].

Nonetheless, the effect of QuickOpt™ interval optimization on the patients' middle- and long-term clinical efficacy remains unclear. To provide reference to the clinicians engaged in CRT-P/D interval optimization, it is proposed to conduct a randomized, double-blind, multicenter and parallel control study in China to observe the effect of QuickOpt™ optimization method on the efficacy in patients at one year post-CRT-P/D implantation compared with echocardiographic optimization. Moreover, the clinical improvement of CRT-P/D patients after standard and regular optimization will be assessed.

1.3 Left ventricular (LV) remodeling

Biventricular pacing could improve the LV remodeling, including the change of LV end-systolic volume (LVESV) and LV end-diastolic volume (LVEDV). The MIRACLE study [4] demonstrated that the indicators of cardiac remodeling were significantly improved one year after CRT-D implantation. In addition, the improvement of LVESV was closely associated with the clinical outcome. Therefore, the decrease in LVESV is often used as an important outcome measure to evaluate whether the patients respond to CRT or not[20].

For that reason, this study takes cardiac remodeling status as the primary endpoint and LVESV as the outcome measure.

1.4 Assessment of body function

Specific Activity Scale (SAS) is a simple method to assess the body function status, which is to grade the exercise capacity in metabolic equivalents (METS) of each individual instead of a simple assessment of quality of life [21-22]. SAS has been used for the clinical trials conducted in regions that are different in cultures and languages, including many studies in North America [23], Australia [24], New Zealand [24], Greece [25], Sweden [26], Denmark [27], Spain [28] and Japan [29-31] so as to assess the treatment response of HF patients. In this study, SAS is used as an outcome measure for body function assessment.

6-Minute Hall Walk Distance (6MHWD) is an outcome measure to detect the exercise tolerance of individuals. In 1985, the study by Guyatt [32] found that 6 MHWD had strong stability and good reproducibility in the evaluation of heart function in CHF patients. Since then there were multiple clinical studies on CHF that evaluated the heart function using 6 MHWD. In the CHF diagnosis and treatment guidelines issued by ACC/AHA in 2002 and 2005, 6-MHWD was specified as a measure for heart function assessment. 6-MHWD has become a common index for the evaluation of heart function of CHF patients now and is generally used for the evaluation of symptom improvement of cardiac function with CRT [33-36]. It is widely used in multiple studies in the world at present.

2 Study objectives

2.1 Purpose

2.1.1 Primary objectives

To demonstrate whether QuickOpt™ can be used as an alternative CRT-P/D optimization method in clinical practice through the comparison of QuickOpt™ optimization with echocardiographic optimization in the clinical improvement in patients at 12 months after CRT-P/D implantation.

(1) To assess the improvement of LV remodeling (LVESV) through QuickOpt™ optimization and echocardiographic optimization in HF patients at 12 months after CRT-P/D implantation.

(2) To assess the change of other clinical manifestations (NYHA class, SAS and 6-MHWD) in above patients with above two different optimization methods.

2.1.2 Secondary objective

To investigate the overall clinical efficacy of CRT-P/D patients receiving regular standard optimization under current medical environment in Mainland China through assessing the clinical improvement in included patients at 12 months after CRT-P/D implantation.

2.2 Study endpoints

2.2.1 Primary endpoint

Cardiac remodeling indicator at 12 months after implantation: LVESV

To minimize the disadvantages of poor reproducibility and large accidental error for echocardiographic measurement, the enrolled patients in each site will be performed with echocardiography by the Echocardiographic Core Laboratory for uniform measurement in this study.

2.2.2 Secondary endpoints

Clinical outcome measures at 12 months after implantation: NHYA class

SAS

6- MHWD

To minimize the effect of the investigators' subjective factors during the assessment of above items, the assessment of these items will be completed by the blinded investigator who will not be informed of the specific optimization method of the patients.

2.2.3 Additional data

Other echocardiographic indexes at 12 months after implantation: LVEF

Mitral regurgitation area

Diagnostic data by programmer: atrium: atrial pacing percentage (%)

AT /AF burden

Times of auto mode switch (AMS)

Ventricle: ventricular pacing percentage (%)

ventricular arrhythmia

Comparison of time consumption between two optimization methods

Drug therapy

Clinical event: emergency, hospitalization, death, etc.

2.3 Screening criteria of patients

2.3.1 Inclusion criteria

The patients can be included into the study only when they meet all the inclusion criteria below:

- Patients with the indications for the implantation of CRT and CRT-P/D who have been implanted with complete lead system (RA lead, RV lead and LV lead) manufactured by SJM
- Able to and willing to receive the regular follow-up as required by this study
- Suitable for drug therapy as appropriate.

2.3.2 Exclusion criteria

The patients who meet any of the following exclusion criteria shall not participate in the study:

- Severe sinus bradycardia (sinus rate≤40bpm)
- Persistent and chronic rapid atrial arrhythmia, including atrial fibrillation, atrial flutter, atrial tachycardia
- II or III degree AV block
- Life expectancy < 1 year
- Age <18 years
- Pregnant and lactating women
- Patients on the treatment of IV positive inotropic drugs
- Patients who are participating in the study of other devices or drugs
- Patients who underwent or plan to undergo heart transplantation
- Hypertrophic obstructive cardiomyopathy
- Patients with severe aortic or mitral stenosis or insufficiency and have not undergone valve replacement
- Patients who underwent coronary artery bypass grafting, percutaneous coronary intervention or cardiomyoplasty within six weeks before inclusion or those with acute coronary syndrome or stroke.
- Patients with pre-excitation syndrome

2.4 Study design

This is a prospective, randomized, double-blind, multicenter (Mainland China), parallel control, non-inferiority, and postmarketing study. The patients who were implanted with CRT-P or CRT-D are randomized to QuickOpt™ optimization group or echocardiographic optimization group in a

1:1 ratio within two weeks after implantation, and followed-up at 3 (±2 weeks), 6 (±2 weeks), and 12 (±2 weeks) months after implantation. In addition to 12 months after implantation, the patients will undergo interval optimization with corresponding method at enrollment, 3 and 6 months, and the optimal AV interval obtained by the optimization are programmed to the patients' CRT-P/D; it is necessary to determine and collect the clinical data at each follow-up.

2.4.1 Study Procedures

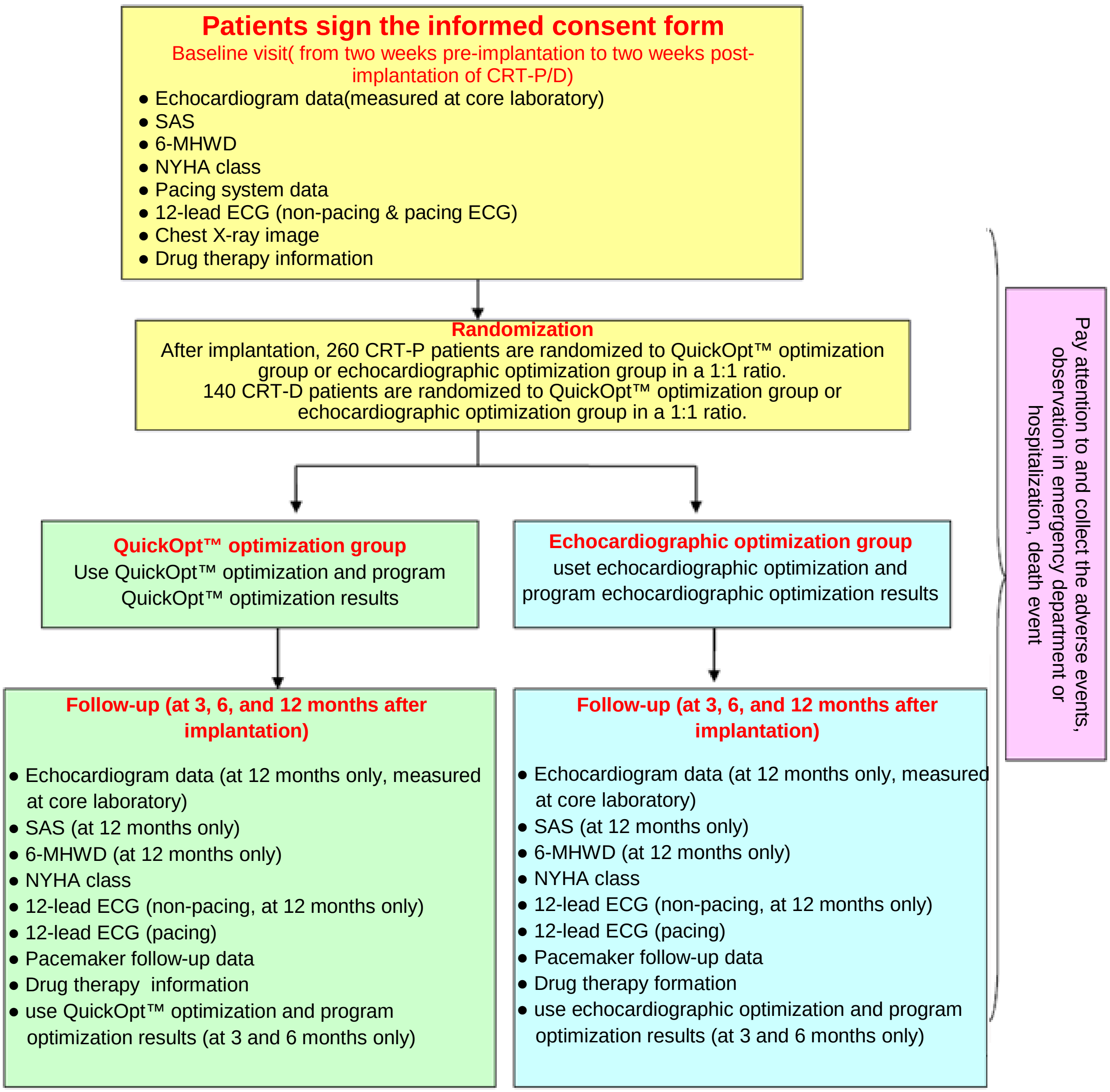

Note:

Unscheduled follow up: In case of any unscheduled follow up for any reason and change of pacing parameters or drug therapy, it is required to fill the unscheduled follow up CRF.

All follow-ups: The optimization results of patients in each group should be used. Otherwise, it is necessary to complete the protocol deviation form and corresponding assessment should be performed. This patient will be followed-up as per the study protocol and included in "intent-to-treat analysis" population for analysis.

Table 1: Data Collection

Tested by	Item	Enrollment	3 months	6 months	12 months	Unscheduled follow up
		Within 2 weeks after operation	(± 2 weeks)	(± 2 weeks)	(± 2 weeks)	Unscheduled
Sampling: Echocardiography technician in each site<br>Measurement: core laboratory	Echocardiogram (DICOM format)	√			√	
Blinded investigator	NYHA class	√	√	√	√	√
	SAS	√			√	
	6 MHWD	√			√	
Investigator not blind to treatment	12-leadECG	Non-pacing & pacing	Pacing	Pacing	Pacing	Non-pacing & pacing
	Chest X-ray image (PA+LAO 45^0)	□√				
	Clinical event*		√	√	√	√
	Pacemaker follow-up**:<br>Printing and retention of the follow-up data<br>Zeroing out: diagnostic data	√***	√	√	√	Diagnostic data not zeroed out
	Interval Optimization	√	√	√		****
	Drug therapy	√	√	√	√	√

Note: *: Emergency, hospitalization, death event, etc. It is necessary to complete corresponding case report form

**: Printing: diagnostic data, programmer test results, final programmed parameters

For QuickOpt™ group, it is necessary to print the test results obtained from QuickOpt™

Storage: CRT-P stores the PDF document of above print contents

CRT-D stores Session Record

***: Program the diagnostic criteria for atrial and ventricular arrhythmia (determined by the investigator based on the clinical conditions) and open AMS

For CRT-P, it is necessary to open the EGM Storage in trigger chamber for rapid ventricular rate

****: The parameters can be adjusted based on the optimization results of different groups.

2.5 Devices used

2.5.1 CRT-P:

Frontier II 5596

2.5.2 CRT-D:

Epic +HF V350
Atlas +HF V341
Atlas II HF V365
Atlas II+ HF V366
Atlas II+ HF V367
Promote 3107-30

Promote 3107-36

2.6 Sample size and duration of the study

The sample size is 400 including 260 for CRT-P and 140 for CRT-D.

The planned enrollment timeis from Q1 in 2010 to December 31, 2011, followed by 1-year follow up and the whole study will be completed in December 2012. There are approximately 40 study sites in Mainland China participating in this study and at least ten patients are estimated to be enrolled in each site.

3 Protocol Description

3.1 Enrollment

The patients should sign the informed consent form before the study.

- The patients who meet all inclusion criteria and do not meet any exclusion criteria can be included.
- The patients who do not meet any inclusion criteria or meet any exclusion criteria cannot be included.

3.2 Baseline visit(within two weeks after implantation)

The entolledpatients are randomized to two groups within two weeks after implantation and, meanwhile, the investigator collects the baseline clinical data.

3.2.1 Randomization

The CRT-P patients are randomized to QuickOpt™ optimization group or echocardiographic optimization group in a 1:1 ratio as per the randomized table with sample size of 260.

The CRT-D patients are randomized to QuickOpt™ optimization group or echocardiographic optimization group in a 1:1 ratio as per the randomized table with sample size of 140.

- QuickOpt™ optimization group: The patients' pacemaker is programmed according to the results of QuickOpt™ optimization (optimized sensing AV interval, optimized pacing AV interval and optimized VV interval). Time of follow-up requiring optimization: baseline (within two weeks after implantation), 3 (±2 weeks) months and 6 (±2 weeks) months; in addition, the investigator should determine whether to optimize interval at unscheduled follow-up.
- Echocardiographic optimization group: The patients' pacemaker is programmed according to the results of echocardiographic optimization (optimized sensing AV interval, optimized pacing AV interval and optimized VV interval). The same as QuickOpt™ optimization group for others. No specific requirement for specific method of echocardiographic optimization is set in this study and the method is determined by each site. The optimization sequence is from AV/PV interval to VV interval based on optimized PV interval. Please refer to the Appendix.

In this study, the patients will not be informed of the randomization to QuickOpt™ or echocardiographic optimization group.

3.2.2 Baseline visit (within two weeks after implantation)

(1) Items in the charge of the clinical investigator:

①Clinical Data

- Basic information of the patients
- 12-lead ECG (non-pacing & pacing)
- Chest X-ray (**PA+LAO 45^0**)
- Information on pacing system implantation
- Pacemaker follow-up: the diagnostic criteria for atrial and ventricular arrhythmia (at

discretion of the investigator) will be programmed and AMS opened

- For CRT-P, it is necessary to open the EGM Storage in trigger chamber for rapid ventricular rate

  Printing: diagnostic data, programmer test results, final programmed parameters

  For QuickOpt™ group, it is necessary to print the QuickOpt™ test results

  Storage: CRT-P stores the PDF of print contents.

  CRT-D stores Session Record

- Zeroing out: diagnostic data zeroed out after printing and storage

- ②Optimization: optimize AV interval according to randomized group and program the optimization results to the pacemaker. If it fails to program based on the optimization results, it is necessary to complete the "protocol deviation form".

Note: If the result of echocardiographic optimization is RV pacing first, to obtain appropriate left AV interval, the result of subtracting the optimized VV interval value from the optimized AV/PV interval is used as the AV/PV interval value for programming. Similarly, for current QuickOpt™, the optimization algorithm is to optimize AV interval and VV interval. Therefore, after QuickOpt™ optimization, if the result is RV pacing first, to obtain appropriate left AV interval, the result of subtracting the optimized VV interval value from the optimized AV/PV interval should be used as the AV/PV interval value for programming.

- Drug therapy

(2) Items in the charge of the blinded investigator:

NYHA class

SAS (refer to the appendix)

6-MHWD (refer to the appendix)

(3) Items in the charge of the echocardiography technician :

- Echocardiogram (stored in the disk in DICOM form to deliver to the core echocardiography laboratory for measurement)

3.3 3 & 6-month follow up after implantation

(1) Time-window: ±2 weeks

(2) Data collection: 12-lead ECG(pacing)

NYHA class (blinding)

Clinical event: emergency, hospitalization, death, etc.

Pacemaker follow-up: the same as the baseline

Change of drug therapy

(3) Optimization: optimize AV interval optimization according to randomized group and program the optimization results to the pacemaker.

QuickOpt™ optimization group: use QuickOpt™ to optimize and obtain the optimization results.

Echocardiographic optimization group: perform echocardiography to optimize and obtain the optimization results.

3.4 12-month follow up after implantation

(1) Time-window: ±2 weeks

(2) Clinical data: echocardiogram data, SAS and 6-MWHD , other data is same as 3 month follow up

(3) Optimization: not required

3.5 Unscheduled follow up

(1) Time window: unscheduled

(2) Clinical data: the same as three months. There is no need to zero out the diagnostic data of the programmer

(3) Optimization: not required. If clinically indicated, it is necessary to optimize according to the randomized group

Note: In case of unscheduled follow up for any reason and change of pacing parameters or drug therapy, it is required to fill the unscheduled follow up CRF.

3.6 Deviation from protocol

The investigator shall comply with the study plan, signed investigator agreement, applicable national or local laws or regulations, and related requirements or provisions of Ethics Committee and CFDA.

Deviation from study protocol refers to a condition where the actual situation is noncompliant with the study plan. The investigator shall inform SJM of any deviation that occurs within 5 working days as soon as possible to avoid threatening the life or health of patients.

3.7 Adverse events

Any adverse medical event occurring from signing the informed consent form by the patients to the end of the study, regardless causal relationship between it and the investigational device, is considered adverse event (AE). Serious adverse event (SAE) refers to any adverse event leads to hospitalization and prolongation of the hospitalization, disability, influence on work capacity, is life-threatening or results in death during clinical study.

According to relevant guidelines in GCP, the investigator is responsible for making medical decisions on the relationship of the event and the clinical study to ensure that the subjects can be appropriately treated in case of any AE during study.

If the AE has no effect on the continuation of study, after the investigator provides appropriate treatment to the subjects, the subjects should continue to receive study treatment until the study is completed except that the subjects withdraw from the study.

The investigator shall classify and report all AEs related to the study protocol according to the severity of AE. All the serious adverse events shall be notified to the CRO by phone as soon as possible (within 24 hours) after awareness and reported to the principal investigator, SJM and relevant Ethics Committee by the CRO. In case of death, the complete follow-up report shall be printed. The common adverse event shall be reported to the CRO within five workdays and the adverse event form shall be completed.

AEs possibly related to CRT-P/D include, but not limited to:
Hematoma
Pneumothorax
Hemothorax
Venous thrombus
Coronary sinus dissection
Coronary sinus perforation
Cardiac perforation
Pericardial tamponade
Infection
Exposed pacing system
Breathing or heartbeat pause
Apnea
Heartbeat pause
Heart failure (deterioration)
Myocardial infarction
Chest wall stimulation
Phrenic nerve or diaphragmatic stimulation
Lead displacement or dislodgement
Lead fracture

Lead insulation damage
Increased pacing threshold
Inadequate sensing
Oversensing
Premature battery depletion

Difficulties in programming or inability to communication or program the pacemaker (i.e. implantation depth, electromagnetic interference or unknown cause)

Pulse generator fault is suspected

Others: all AEs not listed are classified as unexpected events. If the pacemaker, pacing lead system or delivery system causes any AE, SJM will report according to the post-market AE SOP.

3.8 End of study

When the last included patient completes 12-month follow up, the study is ended.

The study cannot be early terminated based on the effectiveness results.

The patient can withdraw from the study at any time for any reason.

4 Statistics

4.1 Sample size

4.1.1 Assumptions

At 12 months after implantation, the patients in QuickOpt™ optimization group shall have the LVESV improvement at least the same as that of the echocardiographic optimization group at the assumed lower limit of -10%.

4.1.2 Calculation of sample size

It is assumed that the significant difference is 0.05 (one-sided), test power is 80%, overall standard derivation is 0.35, and the difference between two group mean is 0.1, the total sample size is 306, with 153 in each group; in consideration of 20% of drop-out rate as a result of death, heart transplantation, loss to follow-up during this study, the total sample size is 382, with 191 in each group. The final total sample size is 400, with 200 in each group.

According to the implantation proportion of CRT-P and CRT-D, for the total 400 patients, it is estimated to include 260 patients for CRT and 140 for CRT-D. The randomized table is generated in a 1:1 ratio for respective sample size.

4.2 Statistical method

t-test will be performed for primary endpoint, LVESV. In case of significantly abnormal distribution and heterogeneity of variance, one of the following non-parametric tests will be performed based on the distribution of final data: Wilcoxon rank sum test, rank test, rank sum test or Kolmogorov-smirnov test.

For secondary endpoint, Cochran-Mantel-Haenszel test will be performed for the comparison of improvement of NYHA class and SAS assessment between these two methods. If the chi-square test indicates homogeneous distribution, Fisher's exact test will be performed. t-test is performed for the difference in the improvement of 6-MHWD between these two methods. In case of significantly abnormal distribution and heterogeneity of variance, one of the following non-parametric tests will be performed based on the distribution of final data: Wilcoxon rank sum test, rank test, rank sum test or Kolmogorov-smirnov test.

5 Risk and Benefit

5.1 Risk

The conventional treatment is performed in the whole study, and the diagnosis and treatment regimen is not changed as a result of the study. In case of any danger, the danger is caused by the conventional treatment instead of the study.

5.2 Benefit

Although the medical costs of included patients will not be exempted as a result of participation, once the patients agree to participate in the study, the physician will pay closer attention to them and their rehabilitation status within one year after operation. In addition, the study is helpful in developing more reasonable therapeutic strategy for patients without affecting the patients' diagnosis and treatment. Moreover, if the patients participate in the study, they can do their bit for the society which may be beneficial to their family members and friends in the future.

6 Study Organization

6.1 *Study management*

6.1.1 Sponsor

This study is sponsored by St. Jude Medical International, Inc. and St. Jude Medical (Shanghai) Co., Ltd.

Contact information of SJM International, Inc.:

The Corporate Village Da Vincilaan 11- box F1 B-1935 Zaventem, Belgium
Tel: (32) 2 774 69 37 Fax: (32) 2 774 69 46

Contact information of SJM China:

St. Jude Medical (Shanghai) Co. Ltd. 20th floor, Xuhuiyuan Mansion, No.1089, 2nd South Zhongshan Road, Xuhui District, Shanghai, 200030, P.R.C
Tel: (86) 21 2306-7588 Fax: (86)21 64224838

6.1.2 Clinical Research Organization (CRO)

The sponsor entrusts Beijing Meihua Easyhin Science and Technology Co., Ltd. (Clinical Research Organization, CRO) with the management of the study project.

B1-403, Building 5, No.9, Chegongzhuang Street, Xicheng District, Beijing
Tel.: 010-88395060-813 Fax: 010-88395070

CRO responsibility:

Scheduling, organization and coordination of clinical study;

Development and management of Electronic Data System used in the study:

Quality supervision and control of project progress;

**On-site inspection:**	**On a regular basis**<br>**Verify the original data and the data on the case report form**<br>**The investigator provides relevant original data to the inspector.**<br>**The investigator and relevant personnel shall actively cooperate with the inspection.**

6.2 *Investigators*

The investigators of this clinical study will be experienced senior investigators from each site. They are senior experts in cardiovascular, pacing and electrophysiological field from national centers who have rich experience in clinical and scientific research and play an important role in clinical and scientific research development in their field.

6.2.1 Principal investigator

The following investigators have been determined as the principal investigators:
Name: 1. Zhang Shu, Professor 2. Huang Dejia, Professor

Hospital: 1. Fuwai Hospital, Chinese Academy of Medical Sciences (CAMS)	2. West China Hospital
Department: 1. Diagnosis and Treatment Center of Arrhythmia	2. Department of Cardiovascular Medicine
Address: 1. No.167, North Lishi Road, Xicheng District, Beijing, China	2. No. 37, Guoxue Alley, Chengdu, Chengdu, China
City: 1. Beijing	2. Chengdu, Sichuan
Country: China	
Tel.: 1. (86) 10 88398843	2. (86) 28 85422343
Fax: 1. (86) 10 68334688	2. (86) 28 85582944

6.2.2 Responsibilities of the investigators

The principal investigators will be responsible for:

- Providing signed investigator/co-investigator agreement;
- Providing the approval document of corresponding Ethics Committee;
- The clinical study should be performed in compliance with the agreement signed with CRO, study plan and all applicable laws and regulations as well as all requirements of the corresponding Ethics Committee or applicable regulatory authority for conducting the study;
- Collecting and retaining the data obtained before implantation, during implantation, at follow-up and after the end of study;
- Strictly following the requirements specified in the study plan to provide the optimal safety and effectiveness of the investigational device applied under clinical conditions; and
- Screening and selecting suitable patients;

It is acceptable that the principal investigator exercises one or more above functions on behalf of the co-investigator or sub-investigator. However, the principal investigator shall also be responsible for correct implementation of clinical study, compliance with the study plan and collection of all the data required. This study shall not be transferred to other implantation center the investigator involves unless otherwise the approval has been obtained from SJM.

In addition to the responsibilities as an investigator, the co-investigator of this study shall also:

- Sign the final version of study plan and the amendment of this study plan;
- Act as the primary contact of all the investigators in case of any medical question during this study.

6.3 Steering Committee (SC)

6.3.1 SC

The steering committee (SC) is the major policy and policy-making body of clinical study and will undertake the most fundamental responsibility of scientific implementation with the principal investigator. SC will, together with the principal investigator, develop the policies and ensure the

scientific value of the project through regular meeting and make a plan to analyze, describe and publish the study results. The members of SC will cooperate with the investigators of each site to, according to the study protocol, complete the enrollment of patients and follow up in time and input the data into the database as soon as possible. According to a failure to comply with the study protocol, inappropriate follow up, inappropriate data entry or AE reporting of each site and other considerations, SC will also be responsible for determining whether to disqualify an medical institution for conducting the study as a site. Each member of SC shall be a well-known expert to ensure the correct implementation of the measures and correct data entry of the study. All investigators participating in the clinical study shall file an application to SC for approval before data analysis, statement or publication.

6.4 Ethical Basis

This study will be carried out in accordance with Declaration of Helsinki of World Medical Association, ISO 14155 and all applicable local laws and regulations. Before this study, this study protocol and informed consent form shall be approved by corresponding Ethics Committee. The copy of the approval document shall be retained by SJM.

6.5 Inspection

This study is sponsored by SJM who entrusts the CRO with the scheduling, organization and coordination of this clinical trial; the development and management of Electronic Data System (EDS) used in this trial; quality monitoring and control during the project; follow-up visit to each site before the end of, during and after this study at appropriate time. As the sponsor, SJM is responsible for ensuring proper monitoring of this study and compliance of progress, records and reports of this study with the clinical study plan, signed clinical study agreement and applicable laws and regulations.

6.5.1 Site initiation

Before this study, the staff of SJM will contact the investigators of the sites to discuss the study plan and view the detailed data requirements. The details and results of the visit will be recorded in the “site initiation” report. Before the initiation of a site, the sponsor shall receive the following documents:

- Copy of approval letter by the Ethics Committee, attached with the copy of approved informed consent form;
- Completed “site initiation” report;
- Signed clinical study agreement;
- Latest resume of the investigators (signed and dated).

6.5.2 On-site inspection

The clinical research associate (CRA) (staff of CRO) will make regular visit to the investigator to supervise and examine the progress of this study and help collect the required data and answer all the questions related to this study. The CRA will ensure the inspection of each site. During each visit, the CRA may view the medical records of patients to verify that all the records and files have been updated and ensure the compliance with the study plan, the applicable national and local laws and regulations and relevant clauses of regulatory authorities. During regular on-site inspection, the investigator shall provide the effective medical records and study records to

the CRA. The CRA shall complete the clinical inspection according to the clinical monitoring procedures.

6.5.3 Inspection of supervision authority (CA)

The investigator and/or CRA shall contact the SJM (Zaventem, Belgium) immediately after receiving the inspection notice of CA. The CRA will assist the investigator in the study file view for the preparation of inspection.

The investigator who has been granted access right shall allow the authorized legal staff to enter into and inspect the facilities (including the facilities where the used devices or records or results are stored) where the devices are stored at a reasonable time in a reasonable way. The investigator or any other personnel on behalf of the investigator shall allow the authorized legal staff to inspect and copy relevant study records at a reasonable time in a reasonable way.

When learning that there is a reasonable suspicion that it fails to obtain appropriate written consent or the investigator fails to submit the required report to the sponsor or EC/IRB, or the submitted report is incomplete, incorrect, false or misleading, the investigator shall allow the authorized legal staff to inspect and copy the records of the subjects.

6.6 Contacts of study management

	**Name**	**Tel.**	**Fax**	**Cellphone**	**Mail**
**Sponsor**	Christophe Bailleul<br>Zhang Nan	+32 27746958<br>+8610 59800600	 +8610 59800660	+33607274016<br>+8613910136390	CBailleul@sjm.com<br>Nzhang@sjm.com
**CRO**	Elizabeth Lau<br>Li Hui	+852 29967609<br>+8610 88395060	+852 2956 0622<br>+861088395070	+852 98800287<br>+8613911012261	elau@sjm.com<br>mmtchina@126.com

Appendix A: Abbreviations

Abbreviation	Term
**6MHWD**	6-Minute Hall Walk Distance
**AF**	Atrial Fibrillation
**AMS**	Auto Mode Switch
**AVD**	Atrioventricular Dislocation
**AVTI**	Aortic Velocity-Time Integral
**BiV**	Biventricular
**CABG**	Coronary Artery Bypass Graft Surgery
**CPL**	Clinical Project Leader
**CRA**	Clinical Research Associate
**CRF**	Case Report Form
**CRT**	Cardiac Resynchronization Therapy
**ECG**	Elctrocardiogram
**EDS**	Electronic Data System
**HF**	Heart Failure
**ICD**	Implantable Cardioverter Defibrillator
**IVCD**	Interventricular Conduction Delay
**IVD**	Interventricular Delay
**kg**	Kilogram
**LBBB**	Left Bundle Branch Block
**LV**	Left Ventricular
**LVEF**	Left Ventricular Ejection Fraction
**ms**	Milliseconds
**mV**	Millivolt
**NA**	Not Applicable
**ND**	Not Done
**NK**	Not Kown
**NYHA**	New York Heart Association
**PCI**	Percutaneous Coronary Intervention
**PG**	Pulse Generator
**PTCA**	Percutaneous Transluminal Coronary Angioplasty
**RA**	Right Atrium
**RBBB**	Right Bundle Branch Block
**RV**	Right Ventricular
**SD**	Standard Deviation
**SJM**	St Jude Medical
**V**	Ventricular
**V**	Volt
**VF**	Ventricular Fibrillation
**VT**	Ventricular Tachycardia

Appendix B: References

1. Cazeau S, et al. Effects of multisite biventricular pacing in patients with heart failure and intraventricular conduction delay. N Engl J Med. 2001; 344:873-80.
2. Abraham WT, et al. Cardiac resynchronization in chronic heart failure. N Engl J Med. 2002; 346:1845-53.
3. Auricchio A, et al. Long-term clinical effect of hemodynamically optimized cardiac resynchronization therapy in patients with heart failure and ventricular conduction delay. J Am Coll Cardiol. 2002; 39:2026- 33.
4. Martin G. St John Sutton, et al. MIRACLE. Circulation. 2006; 113:266-72
5. Bristow MR, et al. COMPANION. N Engl J Med. 2004; 350:2140-50.
6. Cleland JG, et al. CARE-HF, N Engl J Med. 2005 Apr 14; 352:1539-49
7. S. Serge Barold, et al. Echocardiographic optimization of the atrioventricular and interventricular intervals during cardiac resynchronization. Europace. 2008, iii88-95
8. Jeffrey E. Kerlan, et al. Prospective comparison of echocardiographic atrioventricular delay optimization methods for cardiac resynchronization therapy. Heart Rhythm 2006; 3:148-54
9. Phillips KP, et al. Left ventricular resynchronization predicted by individual performance of right and left univentricular pacing: a study on the impact of sequential biventricular pacing on ventricular dyssynchrony. Heart Rhythm. 2007; 4:147-53.
10. Bordachar P, et al. Echocardiographic assessment during exercise of heart failure patients with cardiac resynchronization therapy. Am J Cardiol. 2006; 97:1622-5.
11. Chan, et al. Tissue doppler guided optimization of A-V and V-V Delay of biventricular pacemaker improves response to cardiac resynchronization therapy in heart failure patients. JCF, 2004; 10:S72.
12. Sogaard, et al. Sequential versus simultaneous biventricular resynchronization for severe heart failure: evaluation by tissue Doppler imaging. Circulation 2002; 106:2078-84.
13. O'Donnell, et al. "Long-Term Variations in Optimal Programming of Cardiac Resynchronization Therapy Devices" PACE, 2005; 28:S24-S26.
14. Porciani MC, et al.Temporal variation in optimal atrioventricular and interventricular delay during cardiac resynchronization therapy. J Card Fail 2006; 12:715-9.
15. Valzania C, et al.Cardiac resynchronization therapy: variations in echo-guided optimized atrioventricular and interventricular delays during follow-up. Echocardiography. 2007; 24:933-9.
16. Zhang Q, et al. The role of repeating optimization of trioventricular interval during interim and long-term follow-up after cardiac resynchronization therapy. Int J Cardiol 2008; 124:211-7.
17. James H. Baker II, et al. Acute Evaluation of Programmer-Guided AV/PV and VV Delay Optimization Comparing an IEGM Method and Echocardiogram for Cardiac Resynchronization Therapy in Heart Failure Patients and Dual-Chamber ICD Implants. J Cardiovasc Electrophysiol. 2007; 18:1-7
18. Cinzia Valzania, et al. Cardiac resynchronization therapy during rest and exercise: comparison of two optimization methods. Europace, 2008; 10:1161-69
19. Maria Cristina Porciani, et al, A Real Time 3-Dimensional Echocardiographic Validation of an

Intracardiac Electrogram-Based Method for Optimizing Cardiac Resynchronization Therapy. PACE, 2008; 31:56-63

20. Peter Paul H.M. Delnoy, et al. Sustained Benefit of Cardiac Resynchronization Therapy, J Cardiovasc Electrophysiol, 2007; (18):298-302,
21. Goldman L, et al. Comparative reproducibility and validity of systems for assessing cardiovascular functional class: advantages of a new specific activity scale. Circulation, 1981; 64:1227-34.
22. Lee TH, et al. Estimation of maximum oxygen uptake from clinical data: performance of the Specific Activity Scale. Am Heart J, 1988; 115:203-4.
23. Fleischmann KE, et al. Pacemaker implantation and quality of life in the Mode Selection Trial(MOST).Heart Rhythm, 2006; 3:653-9.
24. Australia-New Zealand Heart Failure Research Collaborative Group. Effects of carvedilol, a vasodilator- beta-blocker, in patients with congestive heart failure due to ischemic heart disease. Circulation, 1995; 92:212-8.
25. Chryssanthopoulos SN, et al. Activity questionnaires; a useful tool in accessing heart failure patients. Int J Cardiol, 2005; 105:294-9.
26. Ekman I, et al. Self-assessed symptoms in chronic heart failure-Important information for clinical management. Eur J Heart Fail. 2007 9: 424-8
27. Kjoller-Hansen L, et al. Effect of ramipril on postrevascularization prevalence of angina and quality of life. Int J Cardiol, 2004; 95:159-65.
28. Jordan AJ, et al. [Assessment of three activity questionnaires in patients with heart failure].Rev Esp Cardiol, 2003; 56:100-3.
29. Effects of pimobendan on adverse cardiac events and physical activities in patients with mild to moderate chronic heart failure: the effects of pimobendan on chronic heart failure study (EPOCH study).Circ J, 2002; 66:149-57.
30. Yasumura Y, et al.Rationale for the use of combination angiotensin-converting enzyme inhibitor and angiotensin II receptor blocker therapy in heart failure.Circ J 2004; 68:361-6.
31. Takeda Y, et al.Effects of carvedilol on plasma B-type natriuretic peptide concentration and symptoms in patients with heart failure and preserved ejection fraction.Am J Cardiol 2004; 94:448-53.
32. Guyatt GH, et al. The 6-minute walk: a new measure of exercise capacity in patients with chronic heart failure. Can Med Assoc J. 1985; 132:919-23
33. Cazeau S, et al. Effects of multisite biventricular pacing in patients with heart failure and intraventricular conduction delay. N Engl J Med, 2001; 344:873-80.
34. Murphy RT, et al. Tissue synchronization imaging and optimal left ventricular pacing site in cardiac resynchronization therapy. Am J Cardiol, 2006; 97:1615-21.
35. Kim WY,et al. Three dimensional echocardiography documents haemodynamic improvement by biventricular pacing in patients with severe heart failure. Heart, 2001; 85:514-20.
36. Garrigue S, et al. Comparison of permanent left ventricular and biventricular pacing in patients with heart failure and chronic atrial fibrillation: prospective haemodynamic study. Heart, 2002; 87:529-34.
