# Supplementary material for: Evaluation of the therapeutic effects of QuickOpt optimization in Chinese patients with chronic heart failure treated by cardiac resynchronization
Source: Sci Rep. 2018 Mar 9;8:4259. doi: 10.1038/s41598-018-22525-0 (PMC5844885; doi:10.1038/s41598-018-22525-0)
Supplement: Supplementary file 3 — Supplementary Datasets [file 41598_2018_22525_MOESM3_ESM.docx]

**Evaluation of the therapeutic effects of QuickOpt optimization in Chinese patients with chronic heart failure treated by cardiac resynchronization**

**Running title**: QuickOpt for CRT-P/D optimization

Ji Yan^1^, Shu Zhang^2#^, Dejia Huang^3#^, Xiaolin Xue^4^, Jing Xu^5^, Qianmin Tao^6^, Weize Zhang^7^, Zheng Zhang^8^, Wei Hua^2^, Yanchun Liang^9^, Baopeng Tang^10^, Wei Xu^11^, Geng Xu^12^, Xuejun Ren^13^, Jingfeng Wang^14^, Tao Guo^15^, Shaobin Jia^16^, Yugang Dong^17^, Hong Jiang^18^, Guosheng Fu^19^, Liguang Zhu^20^, Lin Chen^21^, Fuli Tian^22^, Feng Ling^23^, Jianmei Li^24^, Xiaoyong Qi^25^, Yinglu Hao^26^, Yutang Wang^27^, Liangrong Zheng^6^, Xiaoqun Pu^28^, Farong Shen^29^, Guangping Li^30^, Hui Li^31^, Fang Peng^32^

1. Department of Cardiology, Anhui Provincial Hospital. No 17 Lujiang Road, Hefei 230001, China.
2. Cardiac Arrhythmia center, Fuwai Hospital, Chinese Academy of medical Sciences. No.167 North Lishi Road, Beijing 100037, China.
3. Department of Cardiology, West China Hospital, Sichuan University. No 37 Guoxue lane, Chengdu 610041, China
4. Department of Cardiology, The First Affiliated Hospital of Xi’an Jiaotong University. No 277 Yanta West Road, Xi’an 710061, China.
5. Department of Cardiology, Tianjin chest hospital. No 93 Xi’an Road, Tianjin 300051, China.
6. Department of Cardiology, The First Affiliated Hospital, Zhejiang University. No 79 Qingchun Road, Hangzhou 310003, China
7. Department of Cardiology, The General Hospital of Lanzhou Military. No 98 Xiaoxihu West Street, Lanzhou 730050, China.
8. Department of Cardiology, The First Hospital of Lanzhou University. No 1 Donggang West Road, Lanzhou 730000, China
9. Department of Cardiology, The General Hospital of Shenyang Military. No 83 Wenhua Road, Shenyang 110840, China
10. Department of Cardiology, The First Affiliated Hospital of Xinjiang Medical University. No 137 Liyushan South Road, Urumqi 830054, China
11. Department of Cardiology，Nanjing Drum Tower Hospital, The Affiliated Hospital of Nanjing University Medical School. No 321 Zhongshan Road, Nanjing 210008, China
12. Department of Cardiology, The Second Affiliated Hospital of Zhejiang University School of Medicine. No. 88 Jiefang Road, Hangzhou 310009, China.
13. Department of Cardiology, Beijing Anzhen Hospital, Capital Medical University. No.2 Anzhen Road, Beijing 100029, China.
14. Department of Cardiology, Sun Yat-sen Memorial Hospital of Sun Yat-sen University. No 107 Yanjiang West Road, Guangzhou 510120, China.
15. Department of Cardiology, The First Affiliated Hospital of Kunming Medical University. No 295 Xichang Road, Kunming 650032, China.
16. Department of Cardiology, General Hospital of Ningxia Medical University. No 804 Shengli South Street, Yinchuan 750004, China.
17. Department of Cardiology, The First Affiliated Hospital of Sun Yat-sen University. No 58 Zhongshan Er Road, Guangzhou 510080, China.
18. Department of Cardiology, Renmin Hospital of Wuhan University. No 99 Zhangzhidong Road, Wuhan 430060, China.
19. Department of Cardiology, Sir Run Run Shaw Hospital of Medicine, Zhejiang University No. 3 Qingchun East Road, Hangzhou 310020, China.
20. Department of Cardiology, The First Affiliated Hospital of Guangxi Medical University. No 6 Shuangyong Road, Nanjing 530021, China
21. Department of Cardiology, Fujian Provincial Hospital. No 134 East Street, Fuzhou 350001, China.
22. The First Department of Cardiology, The 251^ST^ Hospital of PLA. No 13 Jianguo Road, Zhangjiakou 075000, China
23. Department of Cardiology, Hangzhou First People's Hospital. No. 261 HuanSha Road, Hangzhou 310006, China
24. Department of Cardiology, The second people’s Hospital of Yunnan Province. No 176 Qingnian Road, Kunming 650021, China
25. Department of Cardiology, Hebei General Hospital. No 348 Heping West Road, Shijiazhuang 050051, China
26. Department of Cardiology, People’s Hospital of Yuxi City. No 21 Nieer Road, Yuxi 653100, China.
27. Department of Cardiology, Chinese PLA General Hospital. No 28 Fuxing Road, Beijing 100853, China
28. Department of Cardiology, Xiangya Hospital Central South University. No 87 xiangya Road, Changsha 410008, China
29. Department of Cardiology, Zhejiang Greentown Hospital. No. 409 Gudun Road, Hangzhou 310000, China.
30. Department of Cardiology, The Second Hospital of Tianjin Medical University. No 23 Pingjiang Road, Tianjin 300211, China.
31. Department of Cardiology, Daqing Oilfield General Hospital. No 9 Zhongkang Street, Daqing 163000, China
32. Department of Cardiology, Shaoxing People’s Hospital. No 568 Zhongxing North Road, Shaoxing 312000, China

**#Corresponding author:**

**#****Shu Zhang**

Cardiac Arrhythmia Center, Fuwai Hospital, Chinese Academy of medical Sciences. No.167 North Lishi Road, Beijing 100037, China.

Tel: +86-13701211342

Fax: +86- 010-68334688

Email: ZhangShu021617@163.com

**#Dejia Huang**

Department of Cardiology, West China Hospital, Sichuan University. No 37 Guoxue Lane, Chengdu 610041, China

Tel: +86- 18980601224

Fax: +86- 028-85422343

Email: HuangDejia021617@163.com

**Supplementary Table 1**: Device implantation information

|  |  | QuickOpt group  N = 198 | Echocardiography group  N = 194 | *P*-value |
| --- | --- | --- | --- | --- |
| Device Type |  |  |  | 0.8407 |
|  | CRTP | 99 (50.0%) | 95 (49.0%) |  |
|  | CRTD | 99 (50.0%) | 99 (51.0%) |  |
| The position of implant |  |  |  |  |
|  | Right side | 1 (0.5%) | 1 (0.5%) |  |
|  | Left side | 197 (99.5%) | 193 (99.5%) |  |
| The positions of the electrodes |  |  |  | 0.4998 |
|  | Great cardiac vein | 1 (0.5%) | 5 (2.6%) |  |
|  | - Posterior vein | 16 (8.1%) | 18 (9.3%) |  |
|  | Posterior lateral veins | 99 (50.0%) | 93 (47.9%) |  |
|  | Lateral veins | 76 (38.4%) | 74 (38.1%) |  |
|  | Middle cardiac vein | 6 (3.0%) | 4 (2.1%) |  |
| The depth of electrodes |  |  |  | 0.2410 |
|  | Proximal segment | 7 (3.5%) | 8 (4.1%) |  |
|  | Middle segment | 129 (65.2%) | 140 (72.2%) |  |
|  | Distal segment | 62 (31.3%) | 46 (23.7%) |  |

**Supplementary Table 2.** Baseline conditions of the patients

| Index | Right atrial lead | | Right ventricular lead | | Left ventricular lead | |
| --- | --- | --- | --- | --- | --- | --- |
|  | QuickOpt^TM^ | Echocardiography group | QuickOpt^TM^ | Echocardiography group | QuickOpt^TM^ | Echocardiography group |
| Pacing threshold (V) | 0.72 ± 0.40 | 0.74 ± 0.44 | 0.64 ± 0.30 | 0.68 ± 0.27 | 1.19 ± 0.67 | 1.32 ± 0.88 |
| Pulse width (ms) | 0.46 ± 0.05 | 0.45 ± 0.05 | 0.46 ± 0.05 | 0.45 ± 0.05 | 0.49 ± 0.17 | 0.48 ± 0.16 |
| P wave amplitude (mV) | 3.44 ± 1.94 | 3.32 ± 1.72 |  |  |  |  |
| Impedance (Ω) | 549.06 ± 126.62 | 553.09 ± 132.78 | 651.81 ± 188.69 | 662.78 ± 208.18 | 853.62 ± 263.59 | 823.31 ± 257.52 |
| R wave amplitude (mV, V=volts) |  |  | 11.40 ± 4.39 | 11.69 ± 4.39 |  |  |
